# Effect of Irradiation and/or Leucocyte Filtration on RBC Storage Lesions

**DOI:** 10.1371/journal.pone.0018328

**Published:** 2011-03-31

**Authors:** Qian Ran, Ping Hao, Yanni Xiao, Jiang Zhao, Xingde Ye, Zhongjun Li

**Affiliations:** 1 Department of Blood Transfusion, Xinqiao Hospital, Third Military Medical University, Shapingba District, Chongqing, China; 2 Oncologic Center, Xinqiao Hospital, Third Military Medical University, Shapingba District, Chongqing, China; University of Sao Paulo – USP, Brazil

## Abstract

Red blood cell (RBC) storage lesions have been shown to be associated with some adverse reactions; numerous studies have focused on the lesions caused by storage, and few data on the RBC storage lesions caused by prestorage treatments of leucocyte filtration and irradiation. In this study, we examined the changes related with the RBC storage lesions, including 2,3-diphosphatidylglyceric acid (2,3-DPG), pH, free hemoglobin (Hb), supernatant free K^+^ and Na^+^ concentration, mean corpuscular volume (MCV), mean corpuscular hemoglobin (MCH). Along with the increasing storage time, decreases in 2, 3-DPG levels, pH and Na^+^ concentration, increases in K^+^ and free Hb concentrations, and significant morphological changes in RBC in all groups were found. The changes in the groups of irradiation, leucocyte filtration and the combined irradiation and leucocyte filtration were more significant than those in the untreated group. Meanwhile, the MCV levels of the three treated groups were significantly lower than those in the untreated group, while the MCH variations were significantly higher. Our results suggest that irradiation and leucocyte filtration before storage may aggravate blood storage lesions.

## Introduction

Blood transfusion administration is a necessary and effective method for clinical surgeries, emergency rescue and hematopathy treatment as well. The influence of blood storage lesions (oxygen free radicals damage erythrocyte membranes, increasing their brittleness and decreasing the transformation index) on blood transfusion treatment has been more valuable. Blood will develop different degrees of damage during storage at 4°C, which will significantly affect the health of patients. Meanwhile, procedures such as irradiation and leucocyte filtration before storage will also lead to different degrees of lesion to red blood cell (RBC) [1]–[2].

Filtration and irradiation brought a series of clinical benefit [3]–[4], such as preventing or delaying the non-haemolytic febrile transfusion reaction (NHFTR), reducing the risk of leukocyte-associated virus transmission (CMV, EBV, HTLV), inhabition the cytokine generation and complement activation, preventing transfusion-associated graft-versus-host disease (TA-GVHD), and so on. Leukoreduction of cellular blood components has become the standard and irradiation has widely applied. Due to the acceleration by filtration and irradiation, the US Food and Drug Administration (FDA) limited the maximal storage time of irradiated RBC to 28 days [5], and 35 days by state food and drug administration (SFDA) in China.

To analyze the RBC lesions variations caused by various blood operations and storage time, we investigated the influence of irradiation and/or leukoreduction by filtration on blood quality by measuring 2,3-diphosphatidylglyceric acid (2,3-DPG) levels, pH, free hemoglobin (Hb), supernatant free K^+^ and Na^+^ concentration, mean corpuscular volume (MCV), mean corpuscular hemoglobin (MCH), mean corpuscular hemoglobin concentration (MCHC) and RBC morphology.

## Results

### RBC morphological changes during storage

**Figure 1:**
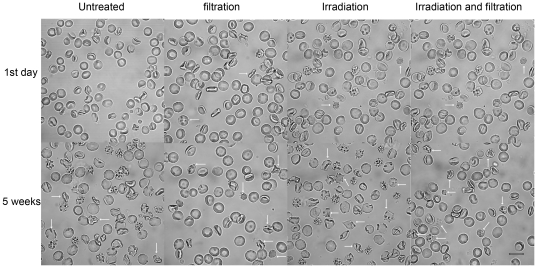
Changes in RBC morphology during storage. Untreated group, leucocyte filtration group, Irradiation group, Irradiation and filtration group after storaged 1 day and 35 days were observed (white arrow indicated deformation and lesion RBC). Images were taken under Zeiss 510 META confocal laser scanning microscopy (original magnification ×1000, scale bar  = 10 µm).

RBC morphology was observed during the storage period using confocal laser scanning microscopy and along with increasing storage time, the normal structure of red blood cells disappeared, and displayed with thin sheet morphology and shrinkage, with a large number of pseudopodia, debris and impurities appearing. This effect was enhanced in all the irradiation and filtration treated groups and treatment was associated with the appearance of a large amount of cell debris (Figure 1).

### Changes in Physical and Chemical Indexes in Storage Process

#### Changes in pH 2,3-DPG levels, free Hb concentrations, supernatant free K^+^ and Na^+^ concentration in Storage Process

**Figure 2:**
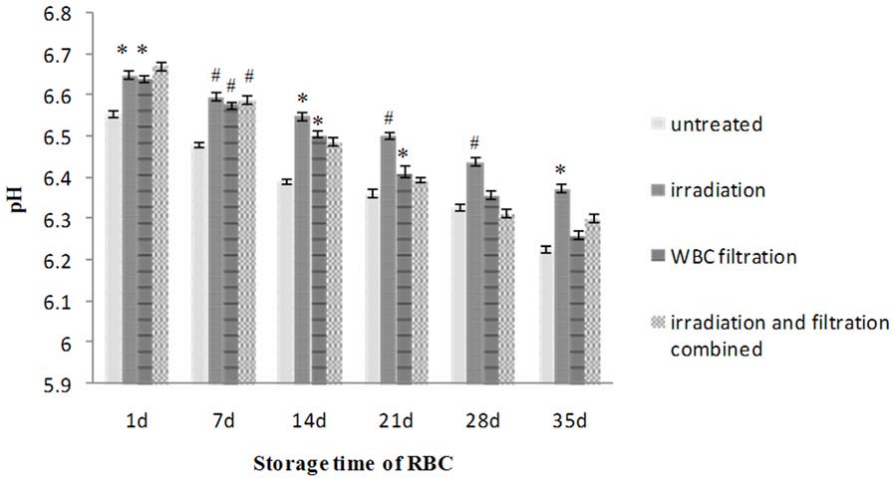
pH changes in each group during storage. #: *P*<0.05 and *: *P*<0.01 compared with untreated group. WBC, white blood cell.

pH was decreased along with the increasing storage time, and was higher in all the treated groups, particularly the irradiation groups, than those of the untreated group (Figure 2).

**Figure 3:**
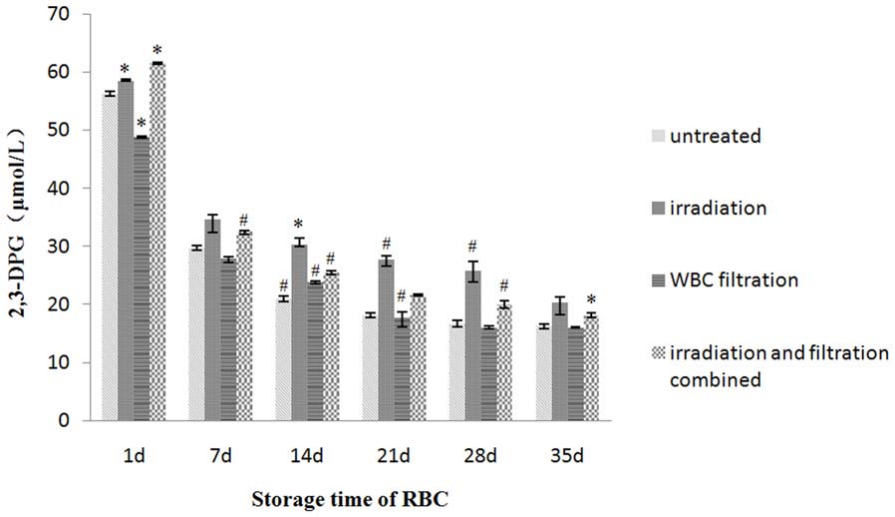
2,3-DPG changes in each group during storage. #: *P*<0.05 and *: *P*<0.01 compared with untreated group.

Along with the increasing storage time, the level of 2,3-DPG in each group decreased. The peak period of observed decrease was 1–7 days, and 21 days later this kind of decrease gradually slowed down (Figure 3). Among the 4 experimental groups, the decrease observed in the filtration group was the most significant, and one day after leucocyte filtration, 2,3-DPG levels dropped to 48.786 µmol/L (85.02%), the lowest one. These changes remained significant between days 1 and 21. Interestingly, the smallest decrease in 2,3-DPG levels in the RBC suspension during storage was observed in the irradiation group.

**Figure 4:**
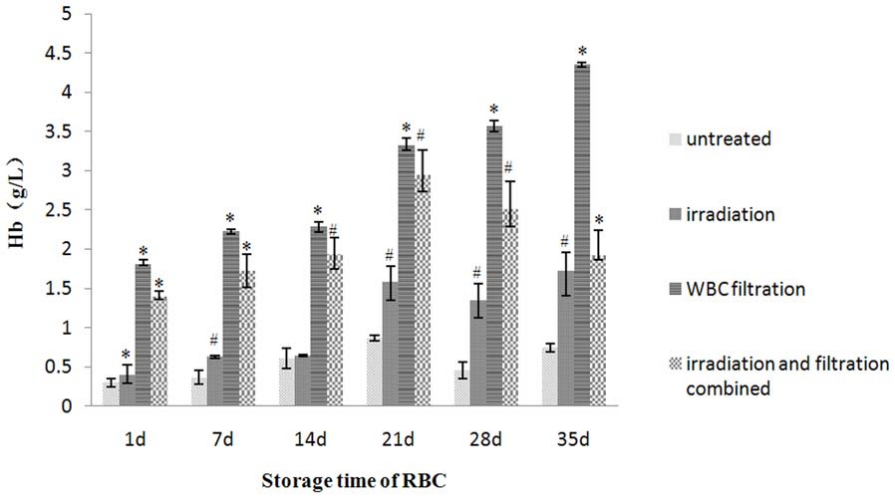
Free Hb concentrations changes in each group during storage. #: *P*<0.05 and *: *P*<0.01 compared with untreated group.

Free Hb concentrations were also increased along with the increasing storage time, and this tendency became more prominent after leucocyte filtration. Compared with the untreated group, the groups after filtration had a rapid and severe increase. Interestingly, after increased to the peak in 21 day, free Hb concentrations in combined irradiation and filtration groups decreased (Figure 4). Changes were seen in all treatment groups at all time points with statistical difference.

**Figure 5:**
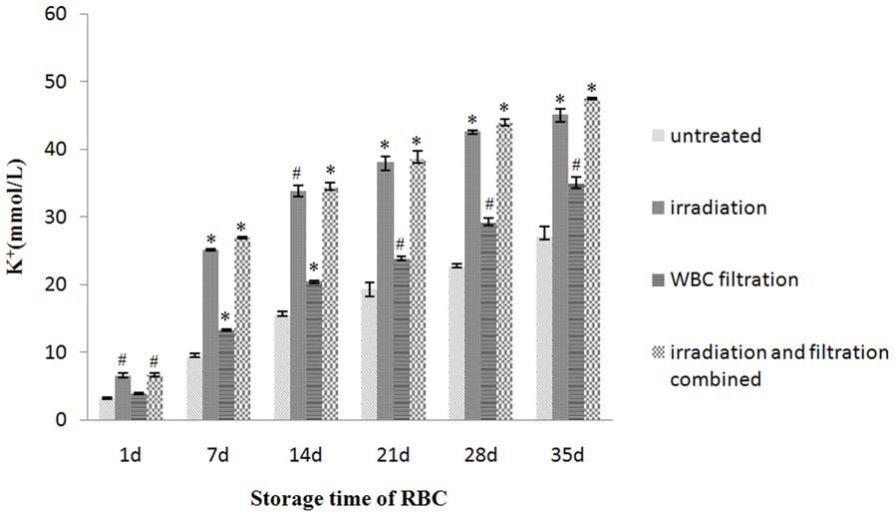
K^+^ concentration changes in each group during storage. #: *P*<0.05 and *: *P*<0.01 compared with untreated group.

Similar to the free Hb concentrations, the K^+^ concentration had a dramatic change after filtration. Although affected slightly after irradiation immediately, the K^+^ concertration of all the samples was increased along with increasing storage time, with the irradiation treatment accelerating the increase in K^+^ concentration. This had increased to over 40 mmol/L after 28 days storage in the filtration and the combined treatment group (Figure 5).

**Figure 6:**
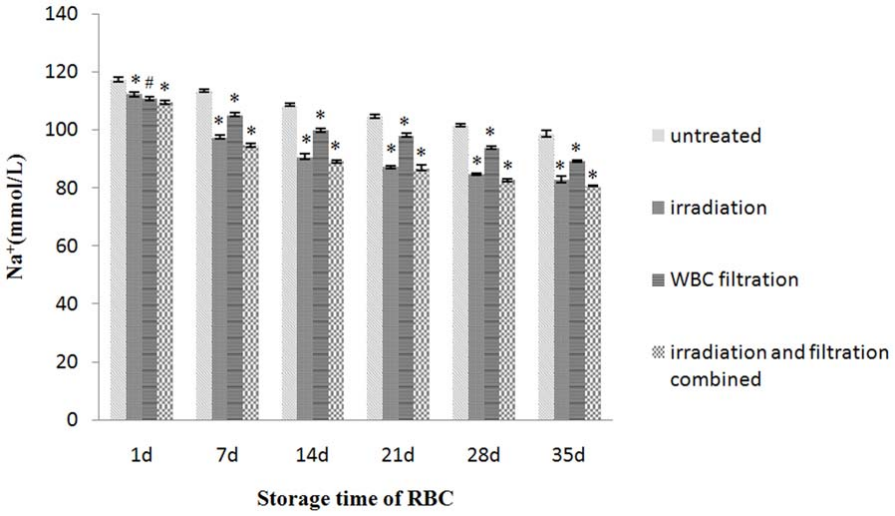
Na^+^ concentration changes in each group during storage. #: *P*<0.05 and *: *P*<0.01 compared with untreated group.

Equally, the Na^+^ concentration of the untreated group, leucocyte filtration group, irradiation group, and combined treatment group was decreased significantly during days 1–14, and on day 14 dropped to 108.30, 90.63, 99.77 and 89.10 mmol/L respectively. But those of the irradiation group and combined treatment group did not decrease significantly after day 14. The Na^+^ concentration on day 28 was 101.53, 84.67, 93.80 and 82.63 mmol/L, respectively (Figure 6).

#### Changes in MCV and MCH during storage

**Figure 7:**
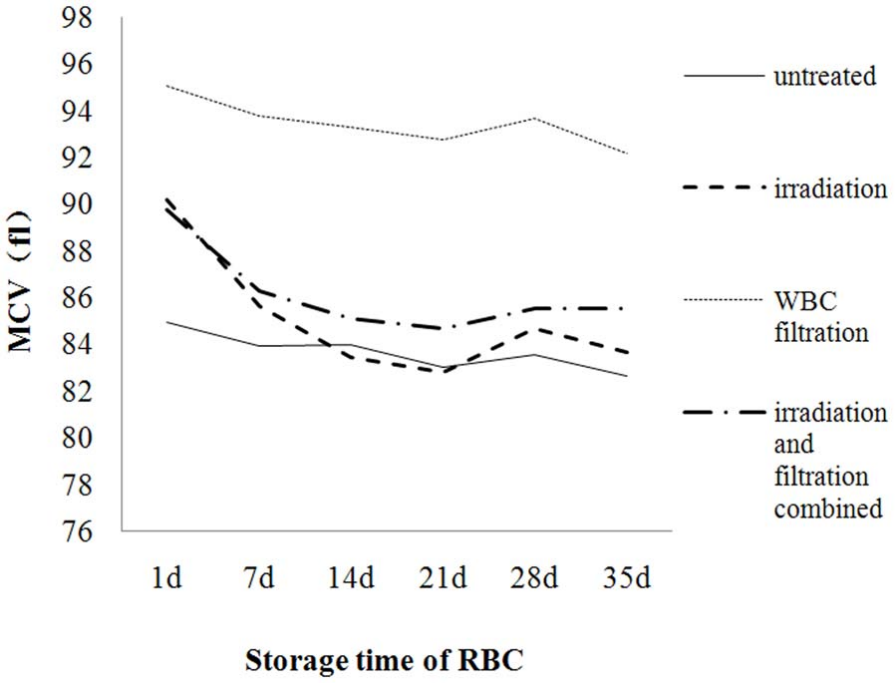
Change in MCV over time during storage at 4°C.

**Figure 8:**
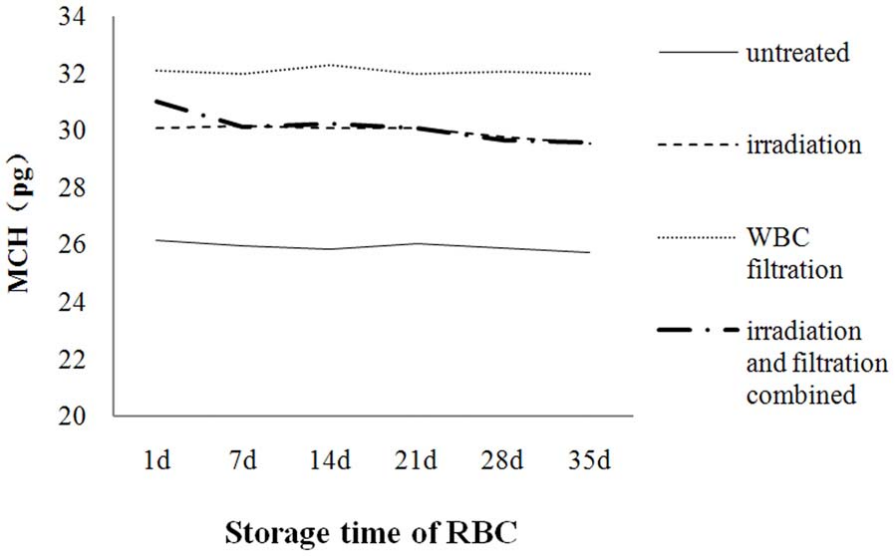
Change in MCH over time during storage at 4°C.

After leucocyte filtration and irradiation, the MCV decreased significantly than that in the untreated group. And the MCH showed a overall downward tendancy during storage, with the MCH of the treated groups higher than than that in the untreated group, but without significance (Figures 7 and 8).

## Discussion

Although RBC transfusion is a life-saving procedure in some circumstances, accumulating evidence links transfusion of RBCs with increased morbidity in certain disease [6]–[7] and mortality in certain patients [8]–[9]. The adverse events are related to the structure and function change of RBCs caused by storage. Numerous studies had focused on the changes of RBC's structure and function caused by storage. Substantial evidences revealed that along with the storage time, cell morphology, potassium concentration, pH, free Hb and 2,3-DPG contents had undergone significant changes [10]–[13]. However, these studies followed with relevant blood-banking industry standard operating procedures, which stored after irradiation and/or filtration, with little research about the relationship between prestorage treatment and RBC storage lesions.

Previous studies had shown that irradiation and filtration resulted in a remarkable alteration of the RBC's properties. Gamma irradiation of plasma caused a significant increase in serum Hb, potassium and LDH, a reduction in deformability and viability [13], [14]. Mechanical force (generated by filtration, such as shear force, pressure and so on) will lead to RBC hemolysis [15]–[16]. Consequently, irradiation and filtration will aggravate RBC storage lesions and accelerate RBC hemolysis in vitro. But, there were little light shed on the influence of RBC storage lesions caused by filtration, irradiation, combined filtration and irradiation.

In this study, we found that irradiation, filtration, combined irradiation and filtration in prestorage can cause significant damage in RBC and intensify the RBC storage lesions. Compared with the untreated group, excluding exceptional cases, irradiation and/or filtration resulted in extremely significant difference in all measured factors (pH, 2,3-DPG, free Hb, K^+^, Na^+^, MCV, MCH). All these changes occurred in very short time (≤1 day). Free Hb concentration showed most obvious increase after irradiation and/or filtration, especially after filtration. The dramatic change in free Hb concentration suggested that filtration in prestorage would induce great damage to the membrane structure, even led to RBCs lesions (mechanical lesions). This lesions might cause cell debris, RBC's intracellular substances and the contents of leucocyte might secondarily damage RBC and worsen RBCs storage lesions. In the storage process, the free Hb, potassium and sodium concentration of all treated groups became increasingly evident, indicating that irradiation and/or filtration in prestorage would intensify RBCs storage lesions in the same storage conditions.

Interestingly, we did observe that after increased to the peak in 21 day, free Hb concentrations in combined irradiation and filtration groups decreased (Figure 4), which seems not consistent with a closed system. As Kriebardis et al. [17] have shown that during storage RBC-derived vesicles contain hemoglobin and many other proteins. We speculated that these RBC-derived vesicles in our system might explain why the free Hb decreased during the storage.

Reverberi et al. [14] suggested that the blood storage before irradiation was inconsequential. Zimmermann et al. [18] also irradiated the leukoreduced blood on day 14 and they showed that there were no significant differences between RBCs irradiated and the ones non-irradiated with respect to the rate of hemolysis, the potasium levels, ATP per g Hb, and 2,3-DPG per g Hb. However, RBC morphology was found to be adversely influenced by storage time and two treatments, with RBC becoming gradually thinner, changing from a full disk and appearing wrinkled and cracked. Previous studies have indicated that morphological changes of RBC, the degradation of hemoprotein and so on, caused RBC to have a lack of variability, elasticity and shortened life cycle [16], [18]–[22]. The life cycle of leucocytes is shorter than 35 days. So, most leucocytes may have already broken down by the end of the 35-day shelf life. The leucocyte debris may not be efficiently filtered and may still cause some adverse reactions during blood transfusion.

Moreover, some severe changes attracted our attention. While on day 28, the potassium concentration of the irradiation group was over 40 mmol/L and the sodium concentration was lower than 85 mmol/L. High potassium concentration may cause acidosis and potassium poisoning. All treated groups had a high concentration of free Hb. This changes could induce an increase in local ADP concentrations and thereby promote thrombosis [23]. A decrease in the 2,3-DPG levels directly increases the affinity of RBC for oxygen, decreases the viability of RBC after infusion, prolongs the time of RBC movement through capillaries, induces inflammation and directly affects the clinical infusion [24]. After long time storage, the 2,3-DPG concentration levels were very low in four groups, suggesting that the function of RBCs was damaged seriously. These changes were associated with adverse reactions after transfusion, and we also found that: TNF-α and IL-6 level of cancer patients had significant changes after transfusion (data not shown).

Our study was the first comprehensive, systematic analysis on the RBC storage lesion caused by irradiation, filtration, combined irradiation and filtration, which is important both to clinical practitioners and to blood bankers. According to the needs of patients to decide whether irradiation and choose the best time to filter would reduce RBC storage lesions. As Reverberi et al. [14] pointed out, probably it is the best practice to irradiate the filtered blood just before transfusion.

In conclusion, our findings have emphasized the importance of the damage to RBC during storage. In particular, irradiation and leucocytes filtration before storage may lead to greater damage during storage, which will directly influence the effectiveness of RBC transfusion. Reducing the adverse transfusion reactions and also maintaining the activity of red blood cell to guarantee an effective transfusion effects is one of the prominent problems that needs to be further studied and resolved.

## Methods

### Prestorage treatments and storage

This study was approved by the Medical Ethnic Review Committee of the Second Hospital Affiliated to the Third Military Medical University. Written informed consents were obtained from all the human subjects involved in this study. Two bags of blood with 200 mL in each were obtained from each voluntary blood donor. Totally 4000 mL blood was taken from 10 donors at the Chongqing Blood Center, and was preserved in RBC preservation solution for the irradiation and/or filtration treatment. Each 1000 ml of RBC preservation solution contained (in mM): 27.5 C_6_H_12_O_6_·6H_2_O, 6.02 NaH_2_PO_4_·2H_2_O, 84.44 NaCl, 80.05 C_6_H_14_O_6_, 1.04 C_5_H_5_N_5_, 5.10 C_6_H_5_O_7_Na_3_·2H_2_O and 0.95 C_6_H_8_O_7_·H_2_O. The irradiation, leucocyte filtration, combined irradiation and filtration were managed on Day 0 and all bags were stored at 4°C. The irradiation, combined irradiation and filtration groups were gamma-irradiated with a dose of 25 Gy by a blood irradiator (BIOBEAM8000, Germany) in Chongqing Blood Center. The irradiation dose was determined using thermoluminescence dosimetry [25]. After the treatments, the blood was randomly divided into 4 groups (untreated, irradiation, leucocyte filtration, irradiation and filtration combined; N = 36 in each). After overnight storage, 24 bags of 25 mL blood sample of Day 1 were shipped to our laboratory at 2 to 8°C. The samples of Day 7, 14, 21, 28 and 35 were treated accordingly to the same procedure for Day 1.

### Measurement in vitro

The samples of each time point (Day 1, 7, 14, 21, 28 and 35) were rejuvenated and used for analysis of 2,3-DPG levels, pH, free Hb levels, free K^+^ and Na^+^ concentrations, MCV and MCH. The changes in RBC morphology were observed over time. Free K^+^, Na^+^ concentrations and pH were analyzed by a blood gas analyzer (sysmex kx-21N hematology analyzer); free Hb and 2,3-DPG levels were determined by corresponding commercial kits (Hb determination kit, purchased from Nanjing Jian Cheng Research Institute, the First Branch of Biological Engineering; 2,3-DPG kit, purchased from USCNLIFE); MCV and MCH were measured by a hematology analyzer (SYSMEX-XE2100, Japan); and cell morphological changes were observed by confocal laser scanning microscopy (Zeiss LSM 510 META).

### Statistical analysis

All the data were expressed as mean ± standard deviation (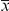 ±s). Student t-test of software SPSS 13.0 was used to analyze the variance among the groups. *P*<0.05 was considered significant different and *P*<0.01 was considered extremely significant different.
